# Differential expression of circulating biomarkers of tumor phenotype and outcomes in previously treated non-small cell lung cancer patients receiving erlotinib vs. cytotoxic chemotherapy

**DOI:** 10.18632/oncotarget.17510

**Published:** 2017-04-28

**Authors:** Mary Jo Fidler, Casey Frankenberger, Richard Seto, Gabriela C Lobato, Cristina L Fhied, Selina Sayidine, Sanjib Basu, Mark Pool, Reem Karmali, Marta Batus, Wen-Rong Lie, David Hayes, Jehangir Mistry, Philip Bonomi, Jeffrey A Borgia

**Affiliations:** ^1^ Section of Medical Oncology, Rush University Medical Center, Chicago, IL 60612, USA; ^2^ Pathology, Rush University Medical Center, Chicago, IL 60612, USA; ^3^ Biochemistry, Rush University Medical Center, Chicago, IL 60612, USA; ^4^ Preventative Medicine, Rush University Medical Center, Chicago, IL 60612, USA; ^5^ Hematology, Oncology and Cell Therapy at Rush University Medical Center, Chicago, IL 60612, USA; ^6^ Present address: Division of Hematology and Oncology, Northwestern University, Chicago, IL 60612, USA; ^7^ EMD Millipore Corporation, St. Charles, MO 63304, USA

**Keywords:** biomarker, non-small cell lung cancer (NSCLC), Luminex, erlotinib, epithelial-to-mesenchymal transition (EMT)

## Abstract

**Background:**

The objective of this study was to identify serum biomarkers capable of predicting clinical outcomes in previously-treated NSCLC patients with wild-type for EGFR activating mutations or insufficient tissue for mutation status determination.

**Methods:**

Sixty-six Luminex immunoassays representative of biological themes that emerged from a re-analysis of transcriptome data from the Cancer Genome Atlas (TCGA) were evaluate against pretreatment serum specimens from previously-treated advanced NSCLC patients received either cytotoxic chemotherapy (n=32) or erlotinib (n=79). Known EGFR mutation positive cases were excluded from analysis. Associations of biomarkers with outcome parameters and their differential interaction with treatment for survival outcomes were assessed using multivariate Cox PH analyses.

**Results:**

Our EMT-based transcriptomic analysis revealed a range of biological processes associated with angiogenesis, apoptosis, cachexia, inflammation, and metabolism emerging as those most highly associated with patient outcome. These processes were evaluated via surrogate serum biomarkers. A treatment-biomarker interaction analysis revealed that higher pretreatment levels of c-Met signaling biomarkers (i.e. HGF levels), pro-inflammatory/ pro-cachexia (e.g. IL-8, sIL-2Rα, FGF-2) processes and a pro-angiogenic (e.g. TGF-α, IL-8, VEGF) milieu were associated with inferior survival (HR=0.35, 0.29, 0.58, 0.50, 0.61, 0.45, respectively; all p<0.05) for patients receiving chemotherapy, relative to erlotinib. In contrast, high levels of decoy receptor for IL-1, sIL-1RII, and a high tissue vimentin/E-cadherin ratio were associated with a poor OS (HR=3.78; p=0.00055) in the erlotinib cohort.

**Conclusions:**

Contemporary precision medicine initiatives that pair patient tumor characteristics with the optimal therapy type may maximize the use of agents targeting EGFR in the treatment of NSCLC.

## INTRODUCTION

Epidermal growth factor receptor (EGFR) tyrosine kinase inhibitors (TKI’s) prolonged survival in molecularly unselected advanced non-small cell lung cancer (NSCLC) patients receiving second or third line therapy and in the maintenance setting [1–3]. Recently, however, erlotinib lost its wild type indication based on consistent trends for prolonged progression free survival (PFS) in wild-type EGFR patients treated with second line docetaxel or pemetrexed over EGFR TKIs [4, 5]. Though EGFR TKI's produce their most pronounced effects in patients with EGFR activating mutations [1–3, 6], prolonged survival in the maintenance setting with erlotinib and recent approval of afatinib for the squamous cell population suggests that EGFR-TKI therapy could be beneficial even in the wild type setting [1, 7]. Additionally, Necitumumab, a monoclonal antibody targeting EGFR was recently FDA approved suggesting persistent drug development in this area is ongoing.

As patients with NSCLC are beginning to live longer with better chemotherapies and the advent of immunotherapy, there is the potential for increased exposure to many agents for NSCLC. The Identification of biomarkers predictive of clinical benefit with EGFR TKI's in wild-type EGFR tumors could offer a well-tolerated option for select patients in this setting. A serum based approach is advantageous for this task as it can provide a real-time snap shot of a patient's disease status and avoids the costs and risks associated with repeat biopsies. Precedent for this approach is a mass-spectrometry assay for serum proteins which was found to be prognostic in NSCLC patients and predictive of chemotherapy benefit over erlotinib for those who have a poor risk serum profile, though recent data suggests the Veristrat assay may be of more prognostic value.

The objective of this study was to identify serum biomarkers capable of predicting favorable clinical outcomes on erlotinib versus palliative chemotherapy in previously-treated NSCLC patients with wild-type for EGFR activating mutations or insufficient tissue for mutation status determination. Herein, we considered 66 unique serum biomarkers related to EMT and evaluated their prognostic and predictive value in 111 cases of pretreated NSCLC lacking known EGFR activating mutations. For these studies we hypothesize that tumor phenotype strongly influences tumor progression and cases with strong epithelial character would benefit the most from strategies targeting EGFR.

## RESULTS

### Overall results of patient cohorts

The median PFS and OS of the entire group was 2.4 months and 7.1 months, respectively The median PFS and OS for the erlotinib group were 1.9 and 6.8 months, respectively and the median PFS and OS for the chemotherapy group were 3.0 and 7.6 months, respectively.

### Association of circulating biomarkers with epithelial versus mesenchymal phenotypes

**Figure 1:**
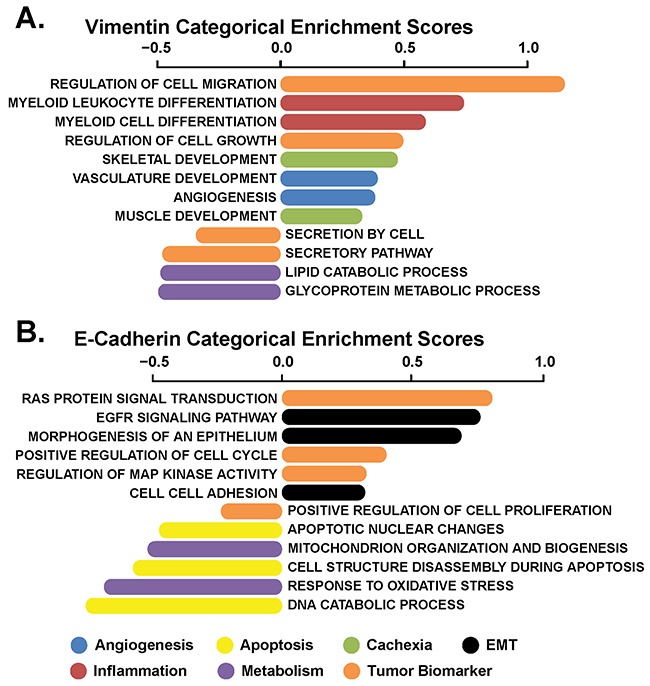
Biological processes enriched in advanced stage NSCLC patients in relation to EMT Categorical analysis of genes profiled from advanced stage NSCLC patients (n=104) that were part of the Cancer Genome Atlas (TCGA), lung adenocarcinoma study (LuAD), whose expression correlates to EMT markers (A) vimentin or (B) E-cadherin. Colors indicate themes of these categories (blue = angiogenesis, yellow = apoptosis, green = cachexia, black = EMT, red = inflammation, purple = metabolism, and orange = tumor biomarker).

**Figure 2:**
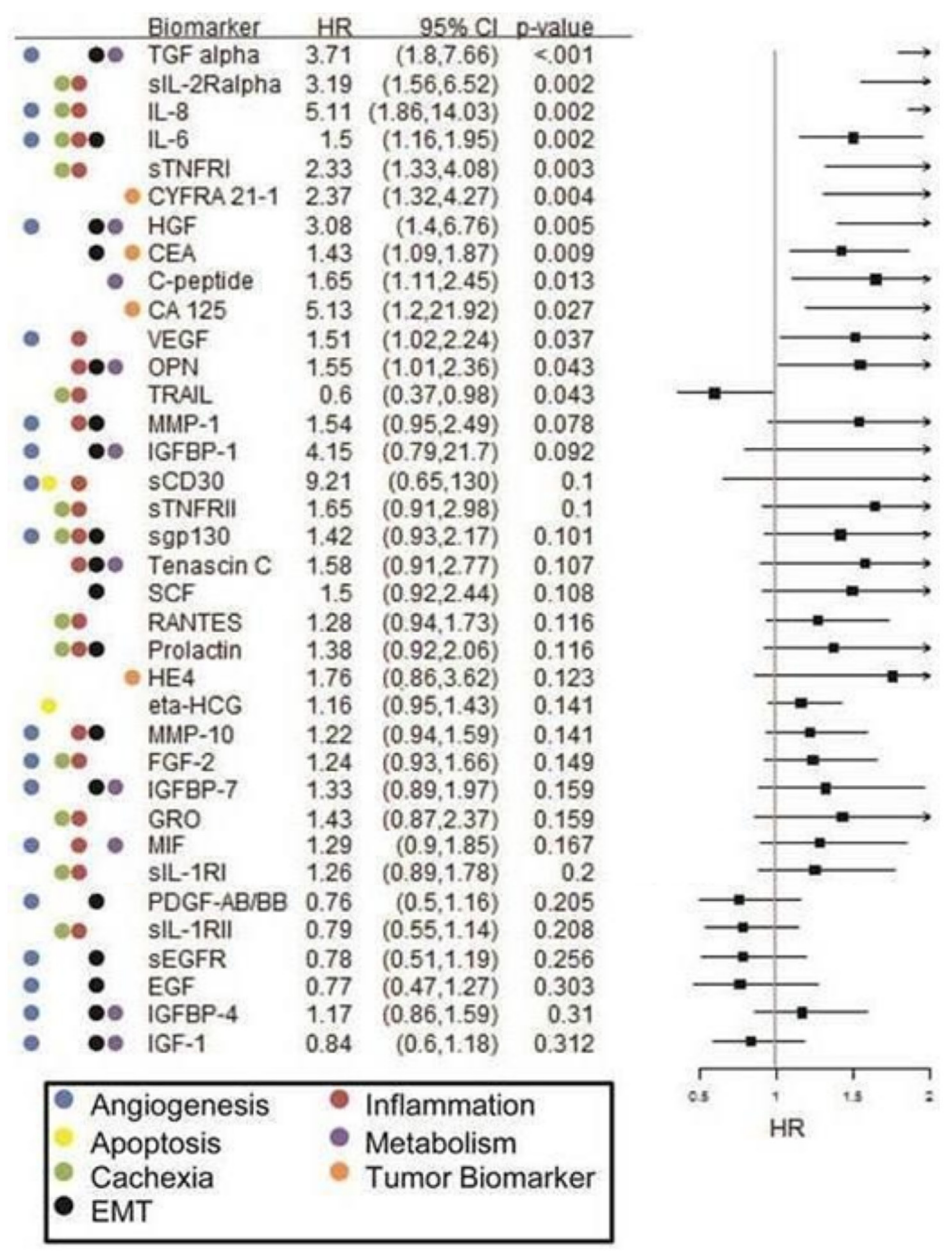
Forest plot of Cox PH regression analysis findings for overall survival in the single-agent chemotherapy cohort Hazard ratios (HR), confidence intervals (CI) and p-values are provided along with examples of known biological pathways/ processes involvement for each biomarker.

**Figure 3:**
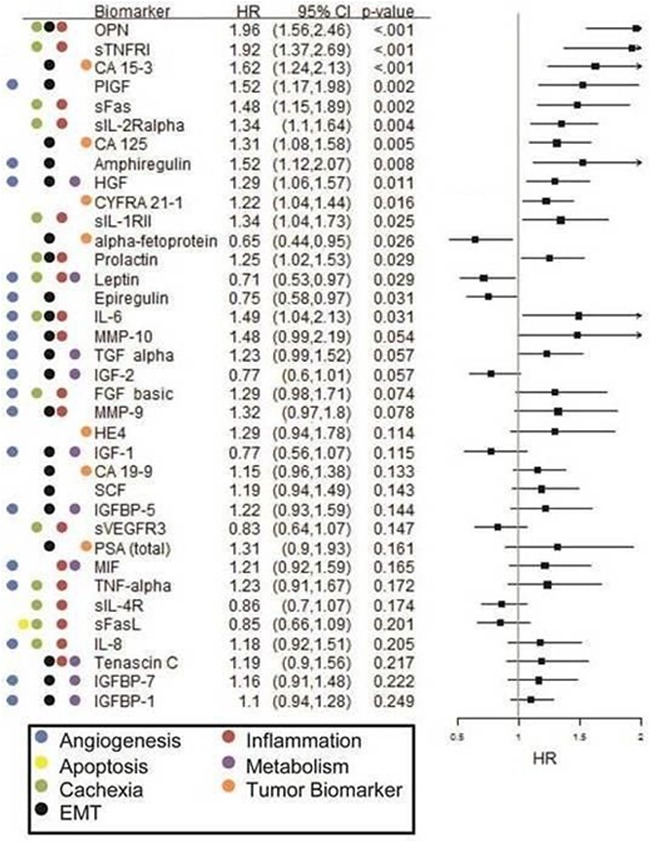
Forest plot of Cox PH regression analysis findings for overall survival in the erlotinib cohort Hazard ratios (HR), confidence intervals (CI) and p-values are provided along with examples of known biological pathways/ processes involvement for each biomarker.

Using transcriptome data from 104 cases of advanced stage NSCLC profiled as part of the Cancer Genome Atlas (TCGA) [8], a gene set analysis (GSA) was performed to reveal biological categories for genes significantly enriched in relation to markers of EMT progression (vimentin vs. E-cadherin expression) (Figures 1A and 1B, respectively). Overall, high levels of biomarkers associated with inflammation (myeloid cell differentiation), angiogenesis (vascular development) and cachexia (skeletal and muscle development) were associated with tumors expressing high vimentin levels, while metabolic processes (lipid catabolism and glycoprotein metabolism) were inversely related to vimentin expression. Furthermore, various gene sets associated with an epithelial phenotype (EGFR signaling, positive regulation of cell cycle, cell-cell adhesion) were enriched in high E-cadherin expressing tumors, while pro-apoptosis (DNA catabolic process, cell structure disassembly during apoptosis, and apoptotic nuclear changes) and metabolism (oxidative stress, biogenesis, and mitochondrial organization) themes were down-regulated. These associations were used, in part, to help guide the selection of circulating biomarkers for the serum studies based on involvement of the biomarkers with processes identified in Figure 1. That is, given increasing mesenchymal character of the tumor is associated with a poor clinical outcome [9–11] as well as increasing vimentin and/or decreasing E-cadherin expression [11–13]; processes associated with these biomarkers should have strong prognostic significance. With this, commercially-available assays of secreted or tumor-shed biomarkers associated with these processes (as indicated in Table 1) were selected for evaluation in the current study. These biomarker categorizations are provided in Figures 2 and 3.

**Table 1:** Demographics and clinicopathological data of patient population based on treatment with erlotinib or Chemotherapy

Characteristic	Erlotinib Patients(n=79)	Chemotherapy Patients(n=32)
**Age**		
*Median*	65.5	63.7
*Range*	(40-88)	(39-82)
**Gender**		
*Male*	33 (42%)	16 (50%)
*Female*	46 (58%)	16 (50%)
**Treatment**		
*Erlotinib*	79	-
*Pemetrexed*	-	21
*Pemetrexed/ cetuximab*	-	3
*Docetaxel*	-	4
*Carboplatin/ Paclitaxel*	-	1
*Gemcitabine/ vinorelbine*	-	1
*Metronomic Cyclophosphamide*	-	1
*Revlamid*	-	1
**Disease Stage**		
*IIIB*	1	5
*IV*	78	27
**Race/ ethnicity**		
*Black*	16	8
*White*	59	24
*Other*	4	-
**Smoking History**		
*Overall*		
*Pack Years*	29	35
*Never*	16	3
*Current/ Former*	63	29
**Histology**		
*Adenocarcinoma*	54 (68%)	16 (50%)
*Squamous Cell*	12 (15%)	6 (19%)
*Other*	13 (17%)	10 (31%)
**Performance Status**		
*0*	19 (24%)	11 (34%)
*1*	50 (63%)	20 (59%)
*2*	8 (10%)	1 (03%)
*3*	2 (3%)	-

Notes: * Definition of smoking history: current smoker or stopped less than 1 year prior to study entry, former smoker, stopped smoking at least 1 year prior to study entry, never smoked or smoked fewer than 100 cigarettes in lifetime.

### Serum biomarker cohort characteristics

The demographics and clinicopathological information on the cohorts are provided in Table 1. Serum samples for proteomic analysis were collected prospectively from 111 patients enrolled in this study. Cases receiving cytotoxic chemotherapy (n=32) were descriptively slightly younger (average 62.7 vs. 65.5 years; p=0.193) than the erlotinib cohort (n=79), with a higher smoking history (35 vs. 29 pack years; p=0.265) and fewer never smokers (3 vs. 16 individuals). Both the chemotherapy cohort and erlotinib groups had approximately equal number of males and females (50% and 58%, respectively). Also, the erlotinib cohort had a slightly higher proportion of patients with adenocarcinoma than the chemotherapy cohort (68% vs. 50%, respectively; p=0.130). In terms of EGFR mutational status, the chemotherapy cohort had 18 (56%) of the cases confirmed negative for mutations, and 14 (44%) cases indeterminate; whereas the erlotinib cohort had 50 (63%) of the cases confirmed negative for mutations and 29 (37%) cases indeterminate due to a lack of evaluable specimens (tumor or plasma). Performance status distributions were balanced between the cohorts, with a majority of patients having Eastern Cooperative Oncology Group (ECOG) performance status of 1. In the subset of patients with immunohistochemical analyses, patients were predominately female 42 (69%); ethnically Caucasian (66%) or African American (30%), had a median smoking history of 35 pack-years (12 never-smokers); and had predominately adenocarcinoma histology (n=42; 69%, squamous cell carcinoma n=8; 13%), unspecified NSCLC (n=11; 18%). 41 of 61 (67%) received erlotinib as their treatment.

### Prognostic value of biomarkers for clinical outcome based on treatment type

#### Chemotherapy cohort

In this cohort, increased pretreatment levels of TGF-α, sIL-2Rα, IL-8, IL-6, sTNF-RI, HGF, and osteopontin were found to be significantly associated with both increased hazard for death (HR= 3.71, 3.19, 5.11, 1.5, 2.33, 3.08, and 1.55, respectively; see Figure 2) as well as increased hazard for progression (see Supplementary Figure 1). [Please note that the HRs represent the impact of one standard deviation change at biomarker level]. Additionally, increased pretreatment levels of CYFRA 21.1, CEA, CA-125, C-peptide, CA-125, and VEGF-A were significantly associated with increased hazard of death (see Figure 2). In contrast, increased levels of TRAIL were significantly associated with decreased hazard of death (HR=0.60; p=0.043) and progression. Altogether, 13 biomarkers were found to have statistically significant (p< 0.05) association with OS and 13 biomarkers were found to have statistically significant (p< 0.05) association with PFS in the chemotherapy cohort based on the Cox PH analyses.

#### Erlotinib cohort

Altogether, 19 biomarkers were found to have statistically significant (p< 0.05) association with OS (Figure 3) and 15 biomarkers were found to have statistically significant (p< 0.05) association with PFS (Supplementary Figure 2) in the erlotinib cohort. Out of these, increased pretreatment circulating levels of amphiregulin, CA-125, CA15-3, osteopontin, PLGF, sFas, sTNF-RI and sTNF-RII were found to be significantly associated with increased hazard of both death as well as progression, whereas increased pretreatment levels of leptin were found to be significantly associated with decreased hazard of both death and progression. Additionally, increased pretreatment levels of sIL-2Rα, HGF, CYFRA 21.1, sIL-1RII, prolactin, and IL-6 were associated (HR=1.34, 1.29, 1.22, 1.34, 1.25, 1.49, respectively) with an increased hazard for death; again, HRs represent the impact of one standard deviation change at biomarker level. In contrast, increased pretreatment levels of α-fetoprotein, leptin, and epiregulin were associated with decreased hazard of death (HR=0.65, 0.71, and 0.75; p=0.026, 0.029, and 0.031; respectively).

### Immunohistochemical analysis

The immunohistochemical analysis of patient-matching tissue specimens (n=61 tested; 58 evaluable) showed a strong association for an increased vimentin to E-cadherin (V/E) ratio with an increased hazard for disease progression or death (HR=1.78 and 2.45; p=0.001 and 0.00024, both respectively) in the erlotinib group (data not shown). E-cadherin, vimentin, or their ratio (V/E) were not found to be significantly associated with progression or death (all p>0.05) in the chemotherapy arm; however, this finding may be reflective of the small number of cases available for this analysis. In the analysis of association of observed biomarker levels with the V/E ratio, high levels of TGF-β, sVEGFR2, MMP-1, sFas, TNF-α, and sIL-6R were found to be positively correlated (Spearman rank correlations, p≤0.05) with increasing V/E ratio. Most commonly, there was co-expression of both epitopes with the pattern of increasing vimentin with decreasing E-cadherin upon adoption of a phenotype with poorer survival statistics. Representative images of immunostaining are shown in Supplementary Figure 3.

### Predictive value of biomarkers using a therapy-biomarker interaction analysis

**Figure 4:**
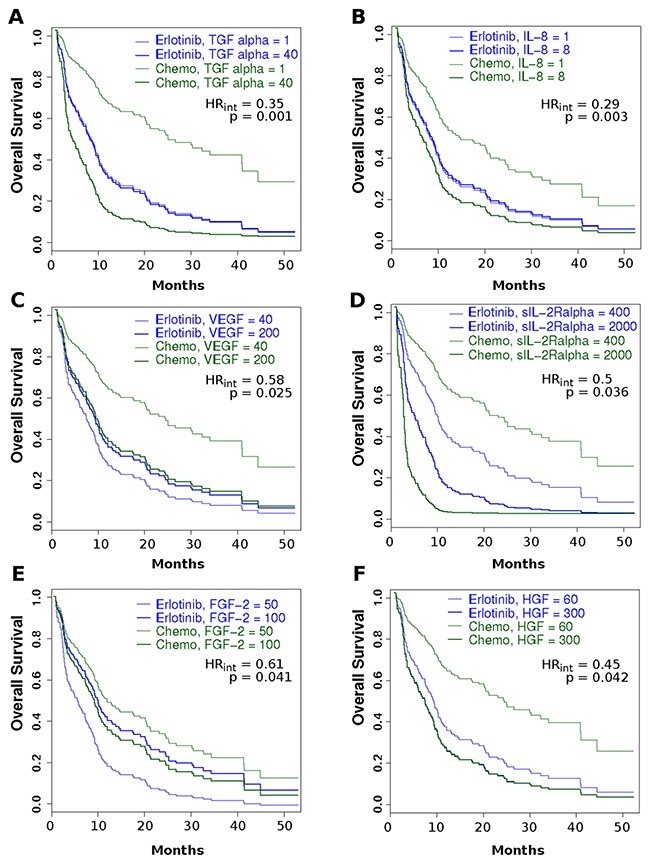
Cox PH interaction model-based predictions of Kaplan-Meier plots for a section of biomarkers Each plot illustrates the overall survival curves based on having either a “low” biomarker level (thin line) or “high” biomarker level (thick line), for patients receiving either erlotinib (blue) or single agent chemotherapy (magenta; “Chemo”). Please note that “low” and “high” levels illustrated here represent the levels observed at the 10^th^ and 90^th^ percentiles for the distribution of measured concentrations for that cohort. Panel A - TGF-α; Panel B – Il-8; Panel C – VEGF; Panel D – sIL-2Rα; Panel E – FGF-2 (aka bFGF); Panel F – HGF. [Example of interpretation: low pretreatment sIL-2Rα levels were associated with a superior outcome in both treatment arms, with the patients receiving chemotherapy performing slightly better than those receiving erlotinib. Those with high levels had a slightly better outcome when erlotinib was administered, relative to patients receiving chemotherapy].

A therapy-biomarker interaction analysis was performed to assess the differential associations of biomarker levels with the hazard of outcome events in the erlotinib cohort relative to those receiving palliative chemotherapy. These differential associations may have value for directing treatment decisions in that they link biomarker levels with clinical outcomes (*i.e*. hazard of outcome events) for erlotinib relative to chemotherapy. In this study we found the therapy-biomarker interaction hazard ratios (of death) for pretreatment levels of TGF-α, IL-8, VEGF, sIL-2Rα, FGF-2, and HGF were all less than 1 (HR = 0.35, 0.29, 0.58, 0.5, 0.61 and 0.45 respectively, all p ≤ 0.05) and only sIL-1RII was found to have a significant interaction HR greater than 1 (HR=1.69; p ≤ 0.05). That is, with increasing levels of TGF-α, IL-8, VEGF, sIL-2Rα, FGF-2, and HGF, the rates of change for hazard of death (in log scale) were found to be lower in the erlotinib group relative to the chemotherapy group or higher for increasing levels of sIL-1RII. These findings are further illustrated in Figure 4, where representative survival plot predictions from the fitted Cox PH Interaction model at the 10^th^ and 90^th^ percentiles of the biomarker values are shown to illustrate survival curves for cases potentially with “low” or “high” biomarker levels. Forest plots of these same data are provided in Supplementary Figure 4, whereas findings for the hazard of disease progression (PFS) are provided in Supplementary Figure 5.

These findings presented above for the therapy-biomarker interaction analysis on the two therapy groups were corroborated with the subgroup analyses reported in Figures 2 and 3. Namely, the HRs (for death) of TGF-α, IL-8, VEGF, sIL-2Rα, and FGF-2 in the chemotherapy cohort were documented as being significant in Figure 2, whereas the HRs for these same markers were not significant in the erlotinib cohort (Figure 3) - leading to a significant differential therapy-biomarker interaction hazard ratio. Additionally, the markedly lower HRs in the erolinib cohort compared to the chemotherapy cohorts for TGF-α (1.23 vs 3.71), IL-8 (1.18 vs 5.11), VEGF (0.88 vs 1.51), sIL-2Rα (1.34 vs 3.19), FGF-2 (0.73 vs 1.24), and HGF (1.29 vs 3.08) further corroborate the inverse HR findings for these markers in the interaction analysis. Note that the subgroup analyses reported in Figures 2 and 3 are respectively on the chemotherapy and erlotinib cohorts, whereas the interactions analysis draws its strength by analyzing the two cohorts combined.

## DISCUSSION

During the EMT process, tumor cells have the potential to adopt certain “cancer stem cell” characteristics, including surface antigens, signaling pathways, and transcriptional regulation mechanisms [14–16]. Perhaps the most archetypal example of EMT features is the loss of E-cadherin expression and up-regulation of alternate adhesion molecules, such as fibronectin or N-cadherin, as well as the intermediate filament protein, vimentin [14, 17]. Our exploratory analysis evaluating patient-matching tissue for E-cadherin (E) and vimentin (V) via immunohistochemistry demonstrated that only in the erlotinib cohort, decreasing E-cadherin and/or increasing vimentin (or a high V/E ratio) were associated with a poor clinical outcome. In other studies this effect was shown to be independent of treatment type [18–20], however, the relatively small size of the chemotherapy group should be considered when interpreting this finding. Our biomarker-interaction test demonstrated that there was a clear increase in hazard of death for patients in the erlotinib cohort, relative to patients receiving single-agent chemotherapy, when the V/E was high. These results support the idea that increasing mesenchymal character in the tumor results in a worse clinical outcome with erlotinib, relative to patients treated with chemotherapy. Moreover, we observed little difference in outcomes for either treatment in patients with high mesenchymal biomarker levels (dark lines, Figure 4), probably resulting from the very influential nature of the tumor processes promoted by this phenotype. However, when these biomarker levels were low (thin lines, Figure 4), very dramatic differences could be observed in outcomes that were dependent on therapy type – with patients receiving chemotherapy being observed to experience an overall better outcome. This finding is consistent with the findings of the TAILOR trial, where EGFR wild-type patient population were documented to have a superior outcome with chemotherapy, versus erlotinib [4]. These combined observations suggest that chemotherapy has greater impact in tumors with an epithelioid phenotype.

Taking this premise further, identifying circulating surrogate influential biomarkers related to EMT would provide an opportunity to evaluate this process in patients for EGFR directed therapy, particularly in wild type patients. For the present study, a Spearman analysis of the observed biomarker levels with the V/E ratio found TGF-β, sVEGFR2, MMP-1, sFas, TNF-α, and sIL-6R as being highly-related variables (p≤0.05) suggesting the potential for serum markers to identify patients that we would expect to have inferior outcomes on EGFR-TKIs. Examination of biomarkers for overall survival identified a series of EMT-associated factors, including osteopontin (OPN), CA15-3, PIGF, CA-125, HGF, prolactin, IL-6, MMP-10, sgp130 and IGFBP-1 with a negative impact on outcome, whereas α-fetoprotein and IGF-2, factors known to oppose EMT processes, were positively associated with outcome in the cohort of 111 patients. The EGFR ligands (amphiregulin, TGF-α, epiregulin, betacellulin, EGF, and HB-EGF) are also known to be intimately involved in promoting EMT, as well as angiogenesis. We observed that amphiregulin, TGF-α, and epiregulin had prognostic values which were consistent with observations made by Vollebergh and colleagues [21]. For example, Figure 4, shows that patients with low levels of TGF-α would have a superior outcome with chemotherapy whereas patients with high levels would benefit more from erlotinib.

Angiogenesis generates tumor neovasculature and is a hallmark of cancer linked to EMT [22]. Through events initiated by low oxygen tension and/or nutrient deprivation caused by tumor cell proliferation [23], hypoxia induced factor-1α (HIF-1α) activation leads to increased gene expression of glucose transporters and glycolytic enzymes, increased expression of pro-angiogenic factors/ decreased expression of anti-angiogenic factors, promotion of adhesion molecule “switching” and metalloproteinase expression that lead to increased cellular motility and invasion that promote metastasis, and enhanced cellular survival/ proliferation [24]. That is, HIF-1α activation initiates the phenotypic changes associated with an epithelial-to-mesenchymal transition (EMT), which have well documented ramifications for promoting EGFR-TKI resistance and poor outcomes [18, 25, 26]. In this study we evaluated a series of factors that promote angiogenesis in lung cancer, including VEGF (VEGF-A), FGF-2, TGF-α, IL-8, PLGF, IGFBP-5, and IL-6 [27–30], as well as a range of matrix metalloproteinases (MMPs), including MMPs -1, -2, -7, and -9, that initiate remodeling of the sub-epithelial stroma and blood vessels for new vessel formation [31, 32]. Our findings were consistent with the idea that high circulating levels of any pro-angiogenic factor have a negative impact on clinical outcomes regardless of treatment (see Figures 2 or 3). However, the pro-angiogenic factors TGF-α, IL-8, VEGF-A, FGF-2, and HGF were found to confer a particularly inferior outcome in the single-agent chemotherapy cohort, as indicated by our therapy-biomarker interaction analysis (see Figure 4 or Supplementary Figure 4) suggesting that erlotinib may possibly mitigate these factors.

Lastly, inflammation is a key promotor of EMT and cancer cachexia and is associated with a poor clinical outcome in lung cancer [33]. This process is thought to be mediated, in part, by circulating inflammatory and/or acute-phase biomarkers [34–37]. We investigated a series of pro- and anti-inflammatory biomarkers as part of this study to determine if these associations were found in our patient cohort and whether biomarker levels were associated with treatment-related outcomes. Not unexpectedly, we observed strong associations of high levels of factors such as IL-6, IL-8, FGF-2, and sIL-2Rα with poor clinical outcomes (see Figures 2 and 3), consistent with documented reports for chronic inflammation and cachexia in multiple cancer types [38–43]. The findings from our biomarker-therapy interaction analysis were provocative with a majority of the significant associations identified being with pro-inflammatory biomarkers. High pretreatment levels of IL-8, sIL-2Rα, and FGF-2 were associated with an increased hazard of death for patients receiving single-agent cytotoxic chemotherapy when compared to those receiving erlotinib. Conversely, high circulating levels of the sIL-1RII were associated with increased hazard of death with erlotinib, relative to those receiving chemotherapy (see Supplementary Figure 4). Interestingly, sIL-1RII is a known anti-inflammatory factor and is thought to function as a circulating “decoy receptor” for IL-1 and IL-1ra, exerting a potent negative effect [44, 45]. High levels of the satiety hormone, leptin, was also associated with an increased hazard of disease progression (HR=0.56; p=0.01) in patients undergoing chemotherapy versus those receiving erlotinib – consistent with the patterns observed with the pro-inflammatory biomarkers.

An obvious weakness of this study is lack of available EGFR mutation data for some of the patients. However, the strength of this paper is that it is studying many circulating biomarkers that signal independent of the EGF receptor that may support preferential use of EGFR targeted therapy in some wild type NSCLC patients. A minor weakness is the lack of post study treatment information though PFS and OS numbers are comparable to published survivals in the previously treated setting and these patients did not have the benefit of exposure to immunotherapy treatments [46]. Our observations require validation. With the recent approval of afatinib and necitumumab in squamous cell NSCLC patients, it would be reasonable to attempt to validate our findings in patients treated with these EGFR inhibitors.

## MATERIALS AND METHODS

### Late stage adenocarcinoma gene set analysis

Gene mRNA expression data and clinical annotation and were downloaded from the Broad Firehose GDAC website [47]. Of 572 lung adenocarcinoma samples, 104 were annotated as being late stage (stage III or stage IV). Using mRNA expression profiles [48] of the late stage adenocarcinoma tumors, gene set analysis was performed using the GSA package (v1.03) in the bioconductor suite of the R statistical package (v3.1.2) [49, 50]. 825 gene sets related to biological processes were tested for enrichment to mRNA expression levels of EMT markers vimentin or E-cadherin in these late stage samples [50, 51]. Two hundred permutations of the response variable were used to establish significance and false discovery.

### Patient population

Patients with stage IV NSCLC that had previously been treated with platinum doublet chemotherapy were enrolled. Serum was collected before initiation of either cytotoxic agents or erlotinib, chosen at the discretion of the treating physician. Serum and clinical data were collected prospectively after written informed consent. Patients were evaluated for disease progression based on version 1.1 of RECIST criteria. The study was performed with written informed patient consent and was approved by the Institutional Review Board at Rush University Medical Center.

EGFR mutational status were determined whenever possible from archival FFPE materials as we previously described [52]. In cases where FFPE materials were not available, EGFR mutational status were determined through digital droplet PCR on cell-free DNA in archived patient plasma or were left as indeterminate from a lack of evaluable specimens. Briefly, circulating free DNA (cfDNA) was purified from 500 μL to 1 mL plasma for each sample using a NucleoSpin Plasma XS kit (Clontech Laboratories, Mountain View, CA) as suggested by the manufacturer. Purity and quantity of the purified cfDNA were examined by NanoDrop (Agilent Technolgies, Santa Clara, CA) and Qubit (ThermoFisher Scientific, Grand Island, NY) instruments independently. Detections of EGFR mutations (G719S and L858R) and exon 19 deletion (E746-A750) in plasma cfDNA by digital droplet PCR were performed in a Bio-Rad QX200 digital PCR System (Bio-Rad Laboratories, Hercules, CA). For each mutation analysis, 10 ng of cfDNA were mixed with 12.5 μL of 2X supermix, 1.25 μL of 20X wild-typed assay and 1.25 μL of 20X mutant assay in a 25 μL reaction. Subsequently, emulsion droplets were generated from the reaction mixture and subjected to PCR amplification. Products of amplification were analyzed by a QX200 Droplet Reader, and data were acquired and analyzed by QuantaSoftTM software (Bio-Rad).

### Collection and storage of serum specimens

Peripheral blood was collected from each patient prior to treatment initiation and processed using standard phlebotomy methods. Briefly, approximately 10 mL of blood was drawn into standard red-top Vacutainers^®^ (without anticoagulant) and permitted to coagulate at room temperature for 30-40 minutes. Following coagulation, the specimens were centrifuged for 15 minutes to yield 4 to 7 mL of sera per tube. Each sera was then spiked with 25 μL/mL of the Mammalian Protease inhibitor cocktail (Sigma, St. Louis, MO) and 10 μL/mL of 0.5M EDTA to prevent further hydrolysis of proteins within the sera. Aliquots of the sera were archived at -80°C in an ultra-low temperature freezer until analysis. No specimen was subject to greater than two freeze-thaw cycles for this study.

### Measurement of serum biomarker concentrations

A total of 66 distinct analytes were evaluated in the Luminex immunobead platform using commercially obtained kits that were executed according to the manufacturer's recommended protocols. All primary data points were collected on a Luminex FLEXMAP 3D^®^ system with concentrations calculated based on 7-point standard curves using a five-parametric fit algorithm in xPONENT^®^ v4.0.3 (Luminex Corp., Austin, TX). Only data that fell within the documented assay range, passed the quality control/ quality assurance measures provided by each kit's manufacturer, and possessed a %CV value ≤10% were considered by our statistician. Any primary read that failed to meet these quality control thresholds were reprocessed until a satisfactory value was obtained.

Analytes were evaluated in the following manner (by kit): IGF-I (MILLIPLEX^®^ MAP Human IGF-I Single Plex; EMD Millipore Corp., Billerica, MA); IGF-II (MILLIPLEX^®^ MAP Cancer Biomarker Panel; EMD Millipore Corp., Billerica, MA); IGFBP-1, IGFBP-2, IGFBP-3, IGFBP-4, IGFBP-5, IGFBP-6, IGFBP-7 (MILLIPLEX^®^ MAP Human IGF Binding Protein (IGFBP) Panel; EMD Millipore Corp., Billerica, MA); C-peptide (MILLIPLEX^®^ MAP Human Endocrine Immunoassay; EMD Millipore Corp., Billerica, MA); sEGFR, sCD30, sgp130, sIL-1RI, sIL-1RII, sIL-2Ralpha, sIL-4R, sIL-6R, sRAGE, sTNFRI, sTNFRII, sVEGFR1, sVEGFR2, sVEGFR3 (MILLIPLEX^®^ MAP Human Soluble Cytokine Receptor Panel; EMD Millipore Corp., Billerica, MA); MMP-1, MMP-2, MMP-7, MMP-9, MMP-10 (MILLIPLEX^®^ MAP Human MMP Panel 2; EMD Millipore Corp., Billerica, MA); TGF-β (MILLIPLEX^®^ MAP Human TGF-β Single Plex; EMD Millipore Corp., Billerica, MA); IL-8, IL-6, GRO, PDGF-AA, PDGF-AB/BB, RANTES (MILLIPLEX^®^ MAP Human Cytokine/ Chemokine Panel I; EMD Millipore Corp., Billerica, MA); HCG, α-fetoprotein, CA-125, CA 15-3, CA 19-9, CEA, HE4, HGF, VEGF, Leptin, MIF, Osteopontin, Prolactin, SCF, sFas, sFasL, TGF-α, TNF-α, Total PSA, TRAIL, CYFRA 21-1 (MILLIPLEX^®^ MAP Human Circulating Cancer Biomarker Panel 1; Amphiregulin, Betacellulin, Epiregulin, EGF, bFGF, HB-EGF, PDGF-BB, PLGF, Tenascin C (Widescreen Human Cancer Panel 2, EMD Millipore Corp.).

### Immunohistochemical studies

A subset of the patients enrolled in the serum studies had evaluable tissue specimens (n=61) from a previous surgical encounter in the tissue archives of our Pathology Department and were further appraised via immunohistochemistry. Formalin-fixed, paraffin-embedded tissue was prepared as five micron sections, transferred to positive-charge slides and baked for 30 minutes at 60 °C. Immunohistochemistry was performed using a Ventana Benchmark autostainer (Ventana Medical Systems, Inc.; Tucson, AZ, USA). Sections were immunostained with either monoclonal mouse anti-vimentin (400-fold dilution; clone V9; DAKO, Carpinteria, CA) or monoclonal mouse anti-E-cadherin (100-fold dilution; clone G-10, Santa Cruz Biotechnology, Santa Cruz, CA). Detection was performed using the iView DAB detection kit, an indirect biotin streptavidin system for detecting mouse and rabbit primary antibodies, on the automated platform per Ventana protocol (Ventana Medical Systems). Staining intensity and distribution for each specimen were recorded in a minimum of 25 randomly selected fields (x400 magnification) along a serpentine pattern using a 3-point intensity-based scoring system, defined as 0, negative; 1, weak; and 2, strong. The percentage of cells staining positive at each of these intensities was recorded for each field evaluated and ultimately averaged to generate values characteristic of the observed staining for each case. A final score was then calculated as the sum of the fractions positive in each of the intensity scores multiplied by the average frequency. Scoring was conducted in a blinded fashion by a minimum of two independent physician observers for each biomarker.

### Biomarker statistical methods

Demographics and clinicopathological data were compared between erlotinib and chemotherapy groups by Mann-Whitney and Fisher exact tests. The associations between time-to-event outcomes (PFS/OS) and biomarkers in continuous scale were assessed by Cox proportional hazards (PH) regression analyses. The biomarkers were analyzed in standardized scales in these PH analyses; thus, the hazard ratios (HRs) resulting from these analyses model the impact of one standard deviation unit change in the biomarker level. The differential impacts of biomarker levels (again in standardized scales) with cytotoxic chemotherapy vs. erlotinib on progression-free survival (PFS) and overall survival (OS) were assessed by multivariate Cox PH interaction model based analyses, using methods similar to those employed in other studies [53, 54].

## SUPPLEMENTARY MATERIALS FIGURES



## References

[1] Cappuzzo F, Ciuleanu T, Stelmakh L, Cicenas S, Szczesna A, Juhasz E, Esteban E, Molinier O, Brugger W, Melezinek I, Klingelschmitt G, Klughammer B, Giaccone G (2010). Erlotinib as maintenance treatment in advanced non-small-cell lung cancer: a multicentre, randomised, placebo-controlled phase 3 study. The Lancet Oncology.

[2] Shepherd FA, Rodrigues Pereira J, Ciuleanu T, Tan EH, Hirsh V, Thongprasert S, Campos D, Maoleekoonpiroj S, Smylie M, Martins R, van Kooten M, Dediu M, Findlay B (2005). Erlotinib in previously treated non-small-cell lung cancer. N Engl J Med.

[3] Thatcher N, Chang A, Parikh P, Rodrigues Pereira J, Ciuleanu T, von Pawel J, Thongprasert S, Tan EH, Pemberton K, Archer V, Carroll K (2005). Gefitinib plus best supportive care in previously treated patients with refractory advanced non-small-cell lung cancer: results from a randomised, placebo-controlled, multicentre study (Iressa Survival Evaluation in Lung Cancer). Lancet.

[4] Garassino MC, Martelli O, Broggini M, Farina G, Veronese S, Rulli E, Bianchi F, Bettini A, Longo F, Moscetti L, Tomirotti M, Marabese M, Ganzinelli M (2013). Erlotinib versus docetaxel as second-line treatment of patients with advanced non-small-cell lung cancer and wild-type EGFR tumours (TAILOR): a randomised controlled trial. The Lancet Oncology.

[5] Lee JK, Hahn S, Kim DW, Suh KJ, Keam B, Kim TM, Lee SH, Heo DS (2014). Epidermal growth factor receptor tyrosine kinase inhibitors vs conventional chemotherapy in non-small cell lung cancer harboring wild-type epidermal growth factor receptor: a meta-analysis. JAMA.

[6] Kawaguchi T, Ando M, Asami K, Okano Y, Fukuda M, Nakagawa H, Ibata H, Kozuki T, Endo T, Tamura A, Kamimura M, Sakamoto K, Yoshimi M (2014). Randomized phase III trial of erlotinib versus docetaxel as second- or third-line therapy in patients with advanced non-small-cell lung cancer: Docetaxel and Erlotinib Lung Cancer Trial (DELTA). J Clin Oncol.

[7] Soria JC, Felip E, Cobo M, Lu S, Syrigos K, Lee KH, Goker E, Georgoulias V, Li W, Isla D, Guclu SZ, Morabito A, Min YJ (2015). Afatinib versus erlotinib as second-line treatment of patients with advanced squamous cell carcinoma of the lung (LUX-Lung 8): an open-label randomised controlled phase 3 trial. Lancet Oncol.

[8] TCGA RN (2014). Comprehensive molecular profiling of lung adenocarcinoma. Nature.

[9] Jakobsen KR, Demuth C, Sorensen BS, Nielsen AL (2016). The role of epithelial to mesenchymal transition in resistance to epidermal growth factor receptor tyrosine kinase inhibitors in non-small cell lung cancer. Transl Lung Cancer Res.

[10] Shintani Y, Okimura A, Sato K, Nakagiri T, Kadota Y, Inoue M, Sawabata N, Minami M, Ikeda N, Kawahara K, Matsumoto T, Matsuura N, Ohta M, Okumura M (2016). Epithelial to mesenchymal transition is a determinant of sensitivity to chemoradiotherapy in non-small cell lung cancer. Ann Thorac Surg.

[11] Soltermann A, Tischler V, Arbogast S, Braun J, Probst-Hensch N, Weder W, Moch H, Kristiansen G (2008). Prognostic significance of epithelial-mesenchymal and mesenchymal-epithelial transition protein expression in non-small cell lung cancer. Clin Cancer Res.

[12] Sabbah M, Emami S, Redeuilh G, Julien S, Prevost G, Zimber A, Ouelaa R, Bracke M, De Wever O, Gespach C (2008). Molecular signature and therapeutic perspective of the epithelial-to-mesenchymal transitions in epithelial cancers. Drug Resist Updat.

[13] Zeisberg M, Neilson EG (2009). Biomarkers for epithelial-mesenchymal transitions. J Clin Invest.

[14] Mani SA, Guo W, Liao MJ, Eaton EN, Ayyanan A, Zhou AY, Brooks M, Reinhard F, Zhang CC, Shipitsin M, Campbell LL, Polyak K, Brisken C (2008). The epithelial-mesenchymal transition generates cells with properties of stem cells. Cell.

[15] Polyak K, Weinberg RA (2009). Transitions between epithelial and mesenchymal states: acquisition of malignant and stem cell traits. Nat Rev Cancer.

[16] Wu Y, Zhou BP (2008). New insights of epithelial-mesenchymal transition in cancer metastasis. Acta Biochim Biophys Sin (Shanghai).

[17] Moreno-Bueno G, Cubillo E, Sarrio D, Peinado H, Rodriguez-Pinilla SM, Villa S, Bolos V, Jorda M, Fabra A, Portillo F, Palacios J, Cano A (2006). Genetic profiling of epithelial cells expressing E-cadherin repressors reveals a distinct role for Snail, Slug, and E47 factors in epithelial-mesenchymal transition. Cancer Res.

[18] Byers LA, Diao L, Wang J, Saintigny P, Girard L, Peyton M, Shen L, Fan Y, Giri U, Tumula PK, Nilsson MB, Gudikote J, Tran H (2013). An epithelial-mesenchymal transition gene signature predicts resistance to EGFR and PI3K inhibitors and identifies Axl as a therapeutic target for overcoming EGFR inhibitor resistance. Clin Cancer Res.

[19] Ren S, Su C, Wang Z, Li J, Fan L, Li B, Li X, Zhao C, Wu C, Hou L, He Y, Gao G, Chen X (2014). Epithelial phenotype as a predictive marker for response to EGFR-TKIs in non-small cell lung cancer patients with wild-type EGFR. Int J Cancer.

[20] Yauch RL, Januario T, Eberhard DA, Cavet G, Zhu W, Fu L, Pham TQ, Soriano R, Stinson J, Seshagiri S, Modrusan Z, Lin CY, O’Neill V, Amler LC (2005). Epithelial versus mesenchymal phenotype determines in vitro sensitivity and predicts clinical activity of erlotinib in lung cancer patients. Clin Cancer Res.

[21] Vollebergh MA, Kappers I, Klomp HM, Buning-Kager JC, Korse CM, Hauptmann M, de Visser KE, van den Heuvel MM, Linn SC (2010). Ligands of epidermal growth factor receptor and the insulin-like growth factor family as serum biomarkers for response to epidermal growth factor receptor inhibitors in patients with advanced non-small cell lung cancer. J Thorac Oncol.

[22] Hanahan D, Weinberg RA (2011). Hallmarks of cancer: the next generation. Cell.

[23] Jackson AL, Zhou B, Kim WY (2010). HIF, hypoxia and the role of angiogenesis in non-small cell lung cancer. Expert Opin Ther Targets.

[24] Lu X, Kang Y (2010). Hypoxia and hypoxia-inducible factors: master regulators of metastasis. Clin Cancer Res.

[25] Ren S, Su C, Wang Z, Li J, Fan L, Li B, Li X, Zhao C, Wu C, Hou L, He Y, Gao G, Chen X (2014). Epithelial phenotype as a predictive marker for response to EGFR-TKIs in non-small cell lung cancer patients with wild-type EGFR. Int J Cancer.

[26] Uramoto H, Yamada T, Yano S, Kondo N, Hasegawa S, Tanaka F (2012). Prognostic value of acquired resistance-related molecules in Japanese patients with NSCLC treated with an EGFR-TKI. Anticancer Res.

[27] Giatromanolaki A, Koukourakis MI, Sivridis E, Turley H, Talks K, Pezzella F, Gatter KC, Harris AL (2001). Relation of hypoxia inducible factor 1 alpha and 2 alpha in operable non-small cell lung cancer to angiogenic/molecular profile of tumours and survival. Br J Cancer.

[28] Minakata K, Takahashi F, Nara T, Hashimoto M, Tajima K, Murakami A, Nurwidya F, Yae S, Koizumi F, Moriyama H, Seyama K, Nishio K, Takahashi K (2012). Hypoxia induces gefitinib resistance in non-small-cell lung cancer with both mutant and wild-type epidermal growth factor receptors. Cancer Sci.

[29] Sanmartin E, Sirera R, Uso M, Blasco A, Gallach S, Figueroa S, Martinez N, Hernando C, Honguero A, Martorell M, Guijarro R, Rosell R, Jantus-Lewintre E, Camps C (2014). A gene signature combining the tissue expression of three angiogenic factors is a prognostic marker in early-stage non-small cell lung cancer. Ann Surg Oncol.

[30] Wan J, Ma J, Mei J, Shan G (2009). The effects of HIF-1alpha on gene expression profiles of NCI-H446 human small cell lung cancer cells. J Exp Clin Cancer Res.

[31] Hadler-Olsen E, Winberg JO, Uhlin-Hansen L (2013). Matrix metalloproteinases in cancer: their value as diagnostic and prognostic markers and therapeutic targets. Tumour Biol.

[32] Loffek S, Schilling O, Franzke CW (2011). Series “matrix metalloproteinases in lung health and disease”: Biological role of matrix metalloproteinases: a critical balance. Eur Respir J.

[33] Laviano A, Koverech A, Mari A (2015). Cachexia: clinical features when inflammation drives malnutrition. Proc Nutr Soc.

[34] Chacon-Cabrera A, Fermoselle C, Urtreger AJ, Mateu-Jimenez M, Diament MJ, de Kier Joffe ED, Sandri M, Barreiro E (2014). Pharmacological Strategies in Lung Cancer-Induced Cachexia: Effects on Muscle Proteolysis, Autophagy, Structure, and Weakness. J Cell Physiol.

[35] Collins J, Noble S, Chester J, Coles B, Byrne A (2014). The assessment and impact of sarcopenia in lung cancer: a systematic literature review. BMJ Open.

[36] Fearon KC, Glass DJ, Guttridge DC (2012). Cancer cachexia: mediators, signaling, and metabolic pathways. Cell Metab.

[37] Kovarik M, Hronek M, Zadak Z (2014). Clinically relevant determinants of body composition, function and nutritional status as mortality predictors in lung cancer patients. Lung Cancer.

[38] Kemik O, Kemik AS, Begenik H, Erdur FM, Emre H, Sumer A, Purisa S, Tuzun S, Kotan C (2012). The relationship among acute-phase responce proteins, cytokines, and hormones in various gastrointestinal cancer types patients with cachectic. Hum Exp Toxicol.

[39] Krzystek-Korpacka M, Diakowska D, Kapturkiewicz B, Bebenek M, Gamian A (2013). Profiles of circulating inflammatory cytokines in colorectal cancer (CRC), high cancer risk conditions, and health are distinct. Possible implications for CRC screening and surveillance. Cancer Lett.

[40] Shibata M, Takekawa M (1999). Increased serum concentration of circulating soluble receptor for interleukin-2 and its effect as a prognostic indicator in cachectic patients with gastric and colorectal cancer. Oncology.

[41] Song B, Zhang D, Wang S, Zheng H, Wang X (2009). Association of interleukin-8 with cachexia from patients with low-third gastric cancer. Comp Funct Genomics.

[42] Watanabe T, Shibata M, Nishiyama H, Soeda S, Furukawa S, Gonda K, Takenoshita S, Fujimori K (2013). Elevated serum levels of vascular endothelial growth factor is effective as a marker for malnutrition and inflammation in patients with ovarian cancer. Biomed Rep.

[43] Watanabe T, Shibata M, Nishiyama H, Soeda S, Furukawa S, Gonda K, Takenoshita S, Fujimori K (2014). Serum levels of rapid turnover proteins are decreased and related to systemic inflammation in patients with ovarian cancer. Oncol Lett.

[44] Gabay C, Lamacchia C, Palmer G (2010). IL-1 pathways in inflammation and human diseases. Nat Rev Rheumatol.

[45] Garlanda C, Dinarello CA, Mantovani A (2013). The interleukin-1 family: back to the future. Immunity.

[46] Shepherd FA (2003). Second-line chemotherapy for non-small cell lung cancer. Expert Rev Anticancer Ther.

[47] Broad Institute TCGA Genome Data Analysis Center: Analysis Overview for Lung Adenocarcinoma (Primary solid tumor cohort).

[48] Li B, Dewey CN (2011). RSEM: accurate transcript quantification from RNA-Seq data with or without a reference genome. BMC Bioinformatics.

[49] Efron B, Tibshirani R (2007). On testing the significance of sets of genes. The Annals of Applied Statistics.

[50] Subramanian A, Tamayo P, Mootha VK, Mukherjee S, Ebert BL, Gillette MA, Paulovich A, Pomeroy SL, Golub TR, Lander ES, Mesirov JP (2005). Gene set enrichment analysis: a knowledge-based approach for interpreting genome-wide expression profiles.

[51] Ashburner M, Ball CA, Blake JA, Botstein D, Butler H, Cherry JM, Davis AP, Dolinski K, Dwight SS, Eppig JT, Harris MA, Hill DP, Issel-Tarver L (2000). Gene ontology: tool for the unification of biology. The Gene Ontology Consortium. Nat Genet.

[52] Buckingham LE, Coon JS, Morrison LE, Jacobson KK, Jewell SS, Kaiser KA, Mauer AM, Muzzafar T, Polowy C, Basu S, Gale M, Villaflor VM, Bonomi P (2007). The prognostic value of chromosome 7 polysomy in non-small cell lung cancer patients treated with gefitinib. J Thorac Oncol.

[53] Scagliotti G, Brodowicz T, Shepherd FA, Zielinski C, Vansteenkiste J, Manegold C, Simms L, Fossella F, Sugarman K, Belani CP (2011). Treatment-by-histology interaction analyses in three phase III trials show superiority of pemetrexed in nonsquamous non-small cell lung cancer. J Thorac Oncol.

[54] Wisnivesky JP, Halm EA (2007). Sex differences in lung cancer survival: do tumors behave differently in elderly women?. J Clin Oncol.

