# Supplementary material for: Differential expression of circulating biomarkers of tumor phenotype and outcomes in previously treated non-small cell lung cancer patients receiving erlotinib vs. cytotoxic chemotherapy
Source: Oncotarget. 2017 Apr 28;8(35):58108–21. doi: 10.18632/oncotarget.17510 (PMC5601637; doi:10.18632/oncotarget.17510)
Supplement: Supplementary file 1 [file oncotarget-08-58108-s001.pdf]

Differential expression of circulating biomarkers of tumor phenotype and outcomes in previously treated non-small cell lung cancer patients receiving erlotinib vs. cytotoxic chemotherapy

SUPPLEMENTARY FIGURES

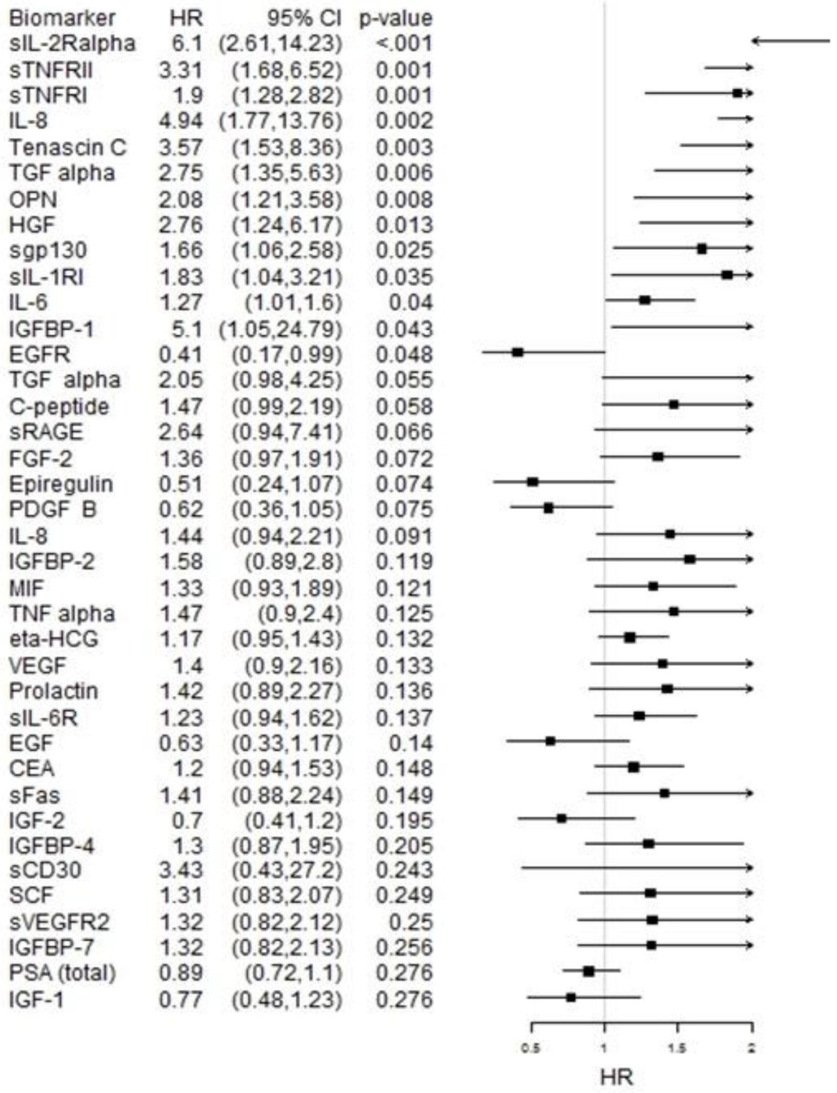

Supplementary Figure 1: Forest plot of Cox PH regression analysis findings for progression-free survival in the chemotherapy cohort. Hazard ratios (HR), confidence intervals (CI) and p-values are provided for each biomarker.

Biomarker	HR	95% CI	p-value
OPN	1.48	(1.19,1.84)	<.001
Leptin	0.6	(0.44,0.81)	0.001
sTNFRII	1.43	(1.16,1.78)	0.001
TGF alpha	1.45	(1.14,1.83)	0.002
Amphiregulin	1.44	(1.11,1.88)	0.007
FGF-2	0.6	(0.41,0.87)	0.008
FGF basic	1.41	(1.09,1.82)	0.008
sTNFRI	1.56	(1.12,2.18)	0.009
CA 15-3	1.87	(1.15,3.05)	0.012
CA 125	1.27	(1.05,1.55)	0.015
IGFBP-1	1.22	(1.03,1.43)	0.018
sFas	1.36	(1.05,1.75)	0.018
PIGF	1.34	(1.04,1.72)	0.023
IGFBP-5	1.31	(1.01,1.71)	0.044
sIL-1RII	1.26	(0.99,1.61)	0.061
IGFBP-2	1.2	(0.99,1.45)	0.063
sIL-4R	0.82	(0.66,1.01)	0.064
sIL-1RI	0.79	(0.62,1.02)	0.069
IL-8	1.26	(0.97,1.64)	0.082
HGF	1.2	(0.97,1.47)	0.087
Betacellulin	0.78	(0.59,1.04)	0.09
IGFBP-4	1.23	(0.96,1.56)	0.096
alpha-fetoprotein	0.73	(0.5,1.06)	0.098
VEGF	1.21	(0.96,1.52)	0.115
sIL-2Ralpha	1.2	(0.96,1.51)	0.116
IGF-2	0.82	(0.63,1.06)	0.133
Prolactin	1.19	(0.94,1.52)	0.143
PDGF B	0.84	(0.66,1.07)	0.155
sRAGE	0.88	(0.72,1.06)	0.171
RANTES	1.17	(0.91,1.5)	0.218
sFasL	0.88	(0.69,1.11)	0.274
sgp130	1.15	(0.88,1.51)	0.304
CYFRA 21-1	1.09	(0.93,1.27)	0.309
PSA (total)	1.21	(0.83,1.77)	0.315
Tenascin C	1.14	(0.88,1.47)	0.324
TNF-alpha	1.16	(0.86,1.57)	0.334
IL-8	0.92	(0.78,1.09)	0.339

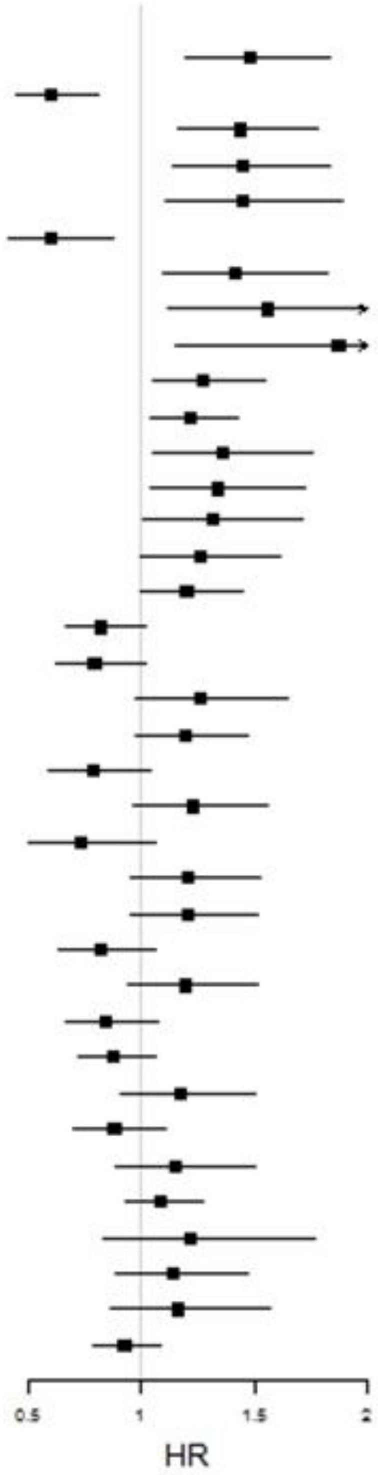

Supplementary Figure 2: Forest plot of Cox PH regression analysis findings for progression-free survival in the erlotinib cohort. Hazard ratios (HR), confidence intervals (CI) and p-values are provided for each biomarker.

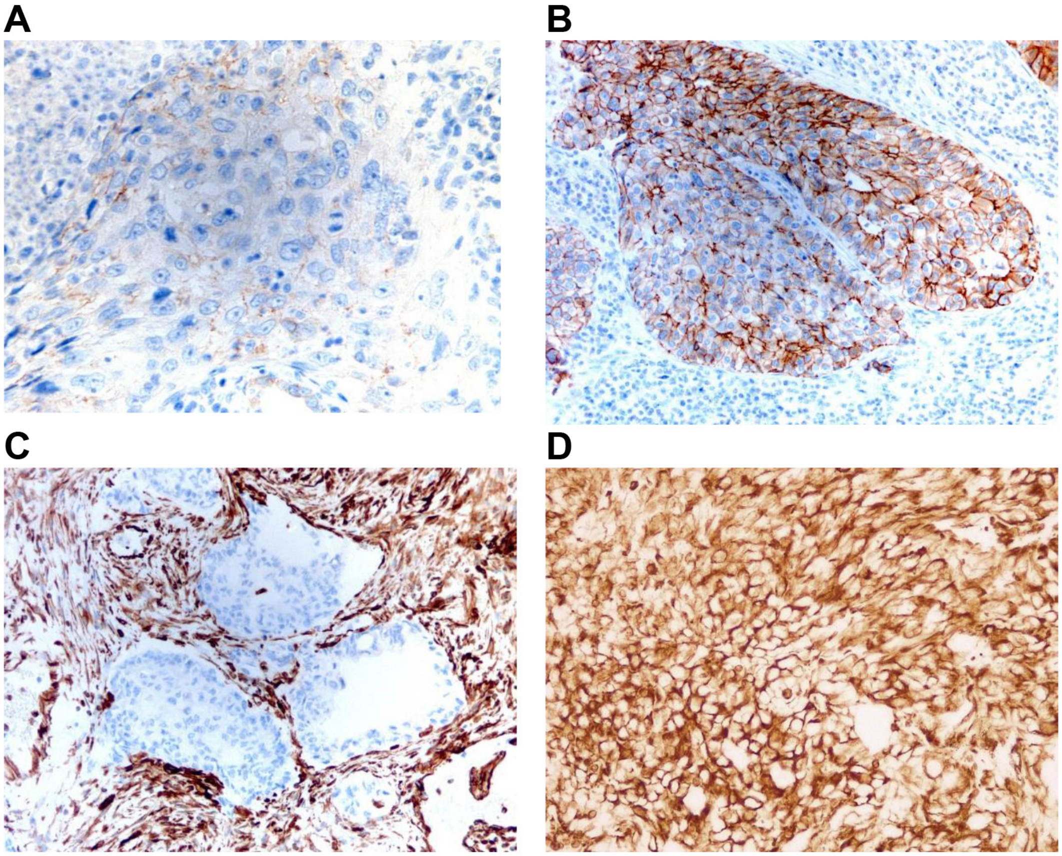

Supplementary Figure 3: Representative immunostaining for E-cadherin and vimentin. Sections demonstrating the typical images for E-cadherin “low” **(panel A)**, E-cadherin “high” **(Panel B)**, vimentin “low” **(Panel C)** and Vimetin “high” **(Panel D)**. Staining intensity and distribution for each specimen were recorded in a minimum of 25 randomly selected fields (x400 magnification) along a serpentine pattern using a 3-point intensity-based scoring system, defined as 0, negative; 1, weak; and 2, strong. The percentage of cells staining positive at each of these intensities was recorded for each field evaluated and ultimately averaged to generate values characteristic of the observed staining for each case. A final score was then calculated as the sum of the fractions positive in each of the intensity scores multiplied by the average frequency.

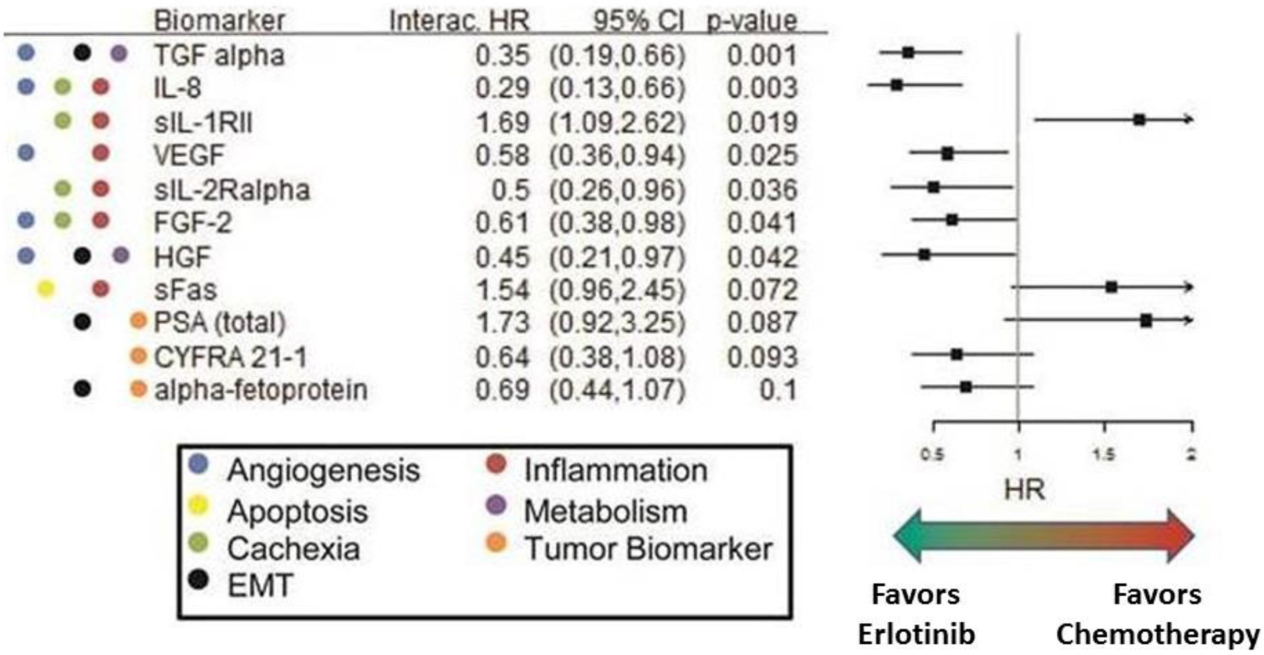

Supplementary Figure 4: Biomarker-Interaction analysis findings presented in forest plot format for overall survival. Hazard ratios (HR), confidence intervals (CI) and p-values are provided along with examples of known biological pathways/ processes involvement for each biomarker. This analysis assigns the differential associations of biomarker levels with the hazard of outcome events in the erlotinib cohort relative to the single-agent chemotherapy patients.

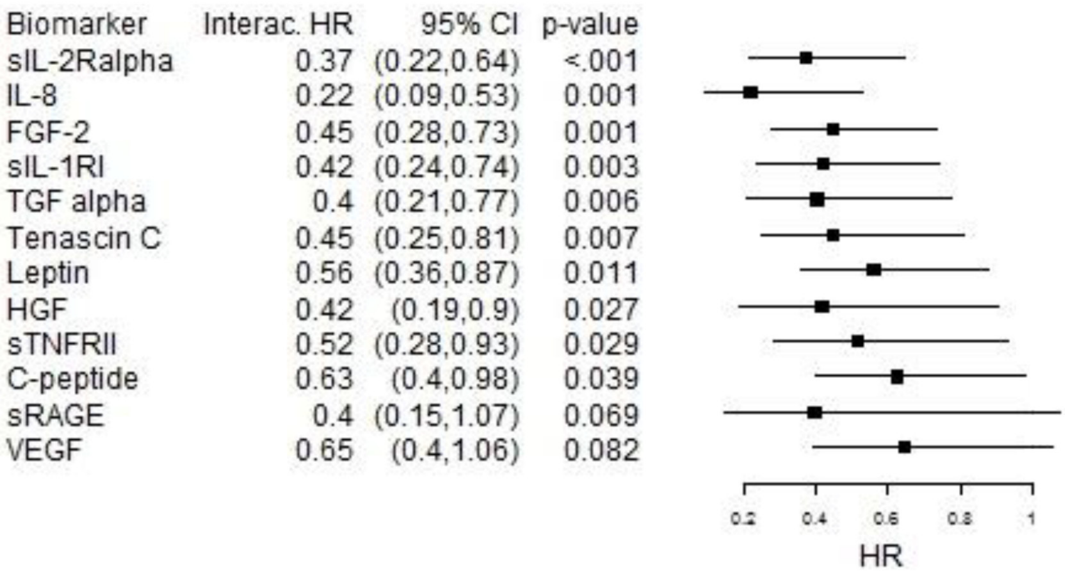

Supplementary Figure 5: Biomarker-Interaction analysis findings presented in forest plot format for progression-free survival. Hazard ratios (HR), confidence intervals (CI) and p-values are provided for each biomarker. High circulating levels of sIL-2Rα, IL-8, FGF-2, sIL-1RI, TGF-α, Tenascin C, leptin, HGF, sTNF-RII and C-peptide were associated (interaction HR=0.37, 0.22, 0.45, 0.42, 0.40, 0.45, 0.56, 0.42, 0.52, and 0.63, respectively; all $p<0.05$) with a relatively higher risk of progression (PFS) in the chemotherapy cohort.
